# *Lonomia obliqua* Venom Induces NF-κB Activation and a Pro-Inflammatory Profile in THP-1-Derived Macrophage

**DOI:** 10.3390/toxins13070462

**Published:** 2021-06-30

**Authors:** Douglas Souza Oliveira, Jean Gabriel de Souza, Miryam Paola Alvarez-Flores, Priscila S. Cunegundes, Carlos DeOcesano-Pereira, Aline Maia Lobba, Renata N. Gomes, Ana Marisa Chudzinski-Tavassi

**Affiliations:** 1 Centre of Excellence in New Target Discovery (CENTD), Butantan Institute, Butantã 05503-900, SP, Brazil; douglas.oliveira@butantan.gv.br (D.S.O.); jean.souza@butantan.gov.br (J.G.d.S.); miryam.flores@butantan.gov.br (M.P.A.-F.); priscila.cunegundes@butantan.gov.br (P.S.C.); carlos.ocesano@butantan.gov.br (C.D.-P.); aline.lobba@butantan.gov.br (A.M.L.); renata.gomes@butantan.gov.br (R.N.G.); 2 Development and Innovation Department, Butantan Institute, Butantã 05503-900, SP, Brazil; 3 Biochemistry Department, Federal University of São Paulo, Vila Clementino 04044-020, SP, Brazil

**Keywords:** *Lonomia obliqua*, inflammation, macrophage activation

## Abstract

Envenomation caused by contact with *Lonomia obliqua* bristles is characterized by pain, an intense systemic proinflammatory reaction and disturbances in the coagulation cascade that can cause severe clinical manifestations and death. However, the role of immune system components in these effects is still poorly understood. In this study, we evaluated the cytotoxic effect of *L. obliqua* venom on THP-1-derived macrophages and its ability to modulate inflammatory markers, as well as the cytokine and chemokine release profile. Our results show that *L. obliqua* venom is able to directly exert a potent pro-inflammatory reaction in macrophages, characterized by the activation of the NF-κB transcription factor pathway, the expression of CD80 and CD83, and the release of pro-inflammatory mediators such as TNF-α, IL-1β, IL-6, IL-8 and CXCL10. These results suggest that macrophages can play an important role during the orchestration of the inflammatory response present in envenomation caused by *Lonomia obliqua* caterpillars.

## 1. Introduction

Caterpillars of the genus Lonomia are present in South and Central America [1,2,3]. *Lonomia obliqua* and *Lonomia achelous* are two species known to cause lonomism, a type of envenomation associated with hemorrhagic syndrome, proinflammatory response and acute renal dysfunction that can lead the victim to death [4].

*L. obliqua*, specifically, is responsible for accidents in the non-Amazonian regions of Brazil, mainly in the Southern Region, and in neighboring countries such as Uruguay, Paraguay and Argentina [5]. Recent data from the epidemiological monitoring direction of Santa Catarina state (Brazil) show that about 200 people were hospitalized for poisoning by *L. obliqua* in 2017 (DIVE—Diretoria de Vigilância Epidemiológica, 2018) [6]. The number of accidents is probably underestimated, since most of the accidents occur in non-urban areas, where it is not always possible to confirm the agent that causes the injuries by the responsible agencies. Thus, lonomism caused by *L. obliqua* is a public health problem in Brazil, and the antilonomic serum produced by the Butantan Institute is the only clinical recourse to revert the dramatic hemorrhagic syndrome in poisoned patients.

Envenomation by the *L. obliqua* caterpillar occurs when victims come into contact with its urticating bristles and the venom is injected subcutaneously. The initial symptoms described after contact with the caterpillar consist of pain, a burning sensation, and an intense local inflammatory reaction, which begins shortly after contact. Severe symptoms appear within 6 to 72 h after contact, such as hemorrhagic diathesis, spontaneous hematomas, bruises, macroscopic hematuria, hematemesis, melena, bleeding of the skin and mucous membranes and generalized hemorrhage [1,7,8]. More severe cases can progress to acute kidney injury or intracerebral bleeding, which are the main causes of death from contact with *L. obliqua* [9,10,11].

Pathophysiologically, the hemorrhage syndrome caused by *L. obliqua* is well characterized [11,12]. It is described in the literature that proteins present in the venom can modulate the victim’s homeostatic system by proteolytically activating coagulation factors, such as prothrombin and X factor, with consequent activation of fibrinogen breakdown and kinine cascades [13,14,15,16,17]. The consumption coagulopathy due to the intensive activation of the coagulation cascade leads to an increase in the concentration of thrombin, plasmin and kallikrein circulating in the blood, which act directly by increasing vascular permeability, inducing hypotension, nociceptive and edematogenic response [11,18,19,20,21].

In addition to the consumption coagulopathy, *L. obliqua* envenomation is characterized by triggering an intense proinflammatory response in the victims, initially manifested by pain and a burning sensation, followed by the formation of edema and erythema [11]. These symptoms are related to disorders in the vascular tissue, intense activation of the immune system and acute renal inflammation [22,23]. In recent years, several studies in vivo and in vitro have been carried out to clarify and relate the role of the inflammatory response induced by the venom in the development of clinical symptoms characteristic of lonomism. Most of inflammatory effects during envenomation rely on the production and release of humoral factors (bradykinin, prostaglandins, histamine), but *L. obliqua* venom proteins have been also proposed to induce the activation of a cellular response that could be involved in the generation and/or amplification of clinical manifestations [16,20,24,25,26].

Macrophages are innate immune cells, important to tissue development, the response to pathogens, surveillance and monitoring changes and the maintenance of tissue homeostasis. Once activated, these cells become specialized phagocytes which engulf and consume cellular debris, foreign bodies and microorganisms, triggering an inflammatory response and tissue remodeling [27]. Macrophages can be activated and polarize into distinct phenotypes based on stimuli and signals from the microenvironment [28]. These cells can polarize into a proinflammatory or anti-inflammatory phenotype, which are characterized by their differences in gene expression, specific membrane markers, metabolic behavior, release of cytokines and biological functions [29,30]. There are two macrophage populations that are well-characterized in vitro, the classically activated macrophages (M1), stimulated by lipopolysaccharides (LPS) and/or proinflammatory cytokines such as IFN-γ, and the alternatively activated macrophages (M2) stimulated with IL-4 and IL-13. M1 macrophages are characterized by the high release of proinflammatory cytokines such as TNF-α, IL-1α, IL-1β, IL-6 and IL-8, and increased expression of MHC II and co-stimulatory molecules, such as CD80 and CD86, which are responsible for inducing complete Th cell activation [31,32]. Moreover, M1 macrophages are characterized by antimicrobial activity and activation of an acute inflammatory response. Meanwhile, alternatively activated macrophages (M2) are characterized by low proinflammatory cytokine production and high production of IL-10 and growth factors such as TGF-β and VEGF [33]. Functionally, M2 macrophages have a potent phagocytosis capacity, promote tissue repair and wound healing, and suppress the proinflammatory response [33,34].

It is still not clear how components of the immune system contribute to trigger the proinflammatory reaction observed in victims of lonomism. Since macrophages are important cells in the triggering, control and resolution of inflammation, the present study evaluated the direct effect of *L. obliqua* crude bristle extract (LOCBE) on THP-1-derived macrophages activation. We showed that *L. obliqua* venom is able to cause the macrophage to take on a proinflammatory phenotype, releasing a considerable amount of TNF-α, IL-1β and IL-6, increasing the expression of activation markers, such as CD80. Moreover, we also showed that the NF-κB pathway may play an important role in these effects.

## 2. Results

### 2.1. LOCBE Cytotoxic Effect on Macrophages

**Figure 1:**
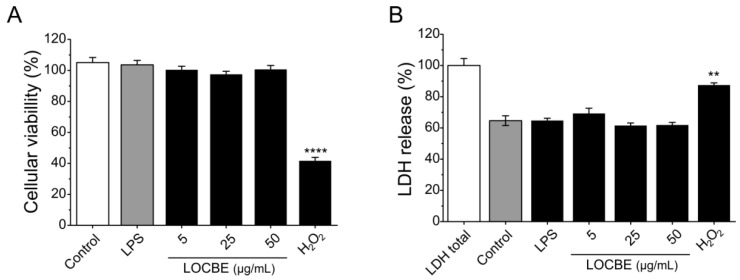
Effect of LOCBE on macrophage viability. (**A**) Cellular viability was measured by MTT assay after 24 h of treatment with three different concentrations of LOCBE. Hydrogen peroxide (H_2_O_2_) was used as the positive control. (**B**) Supernatants from the MTT assay were used to evaluate LDH release as a cytotoxicity marker. All data represent the mean ± S.E.M. Significant differences between the control and treatments were evaluated using Student’s t-test. (*n* = 3, ** *p* < 0.01; **** *p* < 0.0001 vs. control 100%).

No cytotoxicity was detected for macrophages after 24 h in the presence of different concentrations of LOCBE evaluated by MTT and LDH release assays. In the MTT assay, only the positive control, hydrogen peroxide at 1 mM, was able to significantly reduce cell viability by 41.7 ± 2.18% compared to the control (100%). LOCBE treatments in all concentrations assayed caused no effect on cell viability (Figure 1A). A similar result was observed in the LDH release assay (Figure 1B). Additionally, LPS also did not have cytotoxic effect, nor did it induce cell death at the concentration of 1 μg/mL in either of the two assays.

### 2.2. LOCBE Capacity to Activate NF-κB Pathway

**Figure 2:**
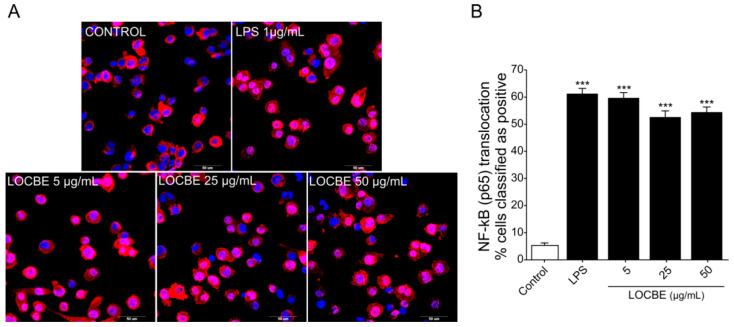
Effect of LOCBE on NF-κB pathway activation. (**A**) Representative images of NF-κB nuclear translocation verified by high content imaging after 30 min of THP1-derived macrophages treatment with LOCBE. Macrophages were fixed and immunostained for NF-κB (p65) antibody and secondary AlexaFluor-647 and the nuclei (blue) were stained with Hoechst 33342 (5 µM). (**B**) NF-κB translocation was calculated by measuring the ratio of fluorescent intensity of the protein in the nucleus and cytoplasm region. Data are expressed as the percentage of positive cells. Negative cells mainly displayed cytoplasmatic staining of NF-κB (red). Stimulation with LPS or LOCBE caused translocation of NF-κB to the nucleus. Data represent the mean ± S.E.M. Significant differences between the control and treatments were performed by Student’s *t*-test (*n* = 3, *** *p* < 0.001).

Nuclear factor-κB (NF-κB) is responsible for regulating pro-inflammatory responses and plays key roles in macrophage survival and polarization [32]. We examined the effect of three different concentrations of LOCBE (5, 25 and 50 μg/mL) on the NF-κB pathway in macrophages (Figure 2A,B). Our data showed that LOCBE was able to induce a significant increase in NF-κB nuclear translocation in THP-1-derived macrophage in all the concentrations tested. While the control group had a basal level of 5.35 ± 0.9% of positive cells, macrophages treated with 5, 25 and 50 μg/mL of LOCBE showed an increase of 59.71 ± 1.95%, 52.65 ± 2.25% and 54.45 ± 1.88% (*n* = 3, * *p* < 0.05 vs. control) of positive cells, respectively. Moreover, there were no statistical differences in LOCBE-induced NF-κB activation when comparing the three concentrations assayed (Student’s *t*-test). The maximum number of positive cells value was obtained in the lowest concentration tested, 5 µg/mL.

### 2.3. LOCBE Upregulates Inflammatory Genes Expression

**Figure 3:**
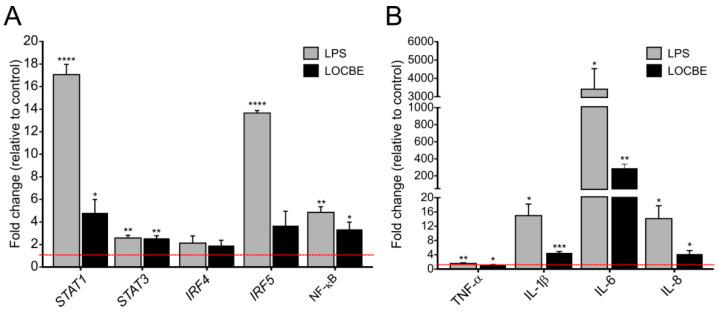
LOCBE upregulates gene expression of inflammatory modulators. mRNA expression levels of transcriptional factors genes related to the regulation of the inflammatory response (*STAT1*, *STAT3*, *IRF4*, *IRF5*, *NF-κB*) (**A**) and proinflammatory cytokine genes (*TNF-α*, *IL-1β*, *IL-6* and *IL-8*) (**B**) in macrophage after 6 h treatment with 5 µg/mL LOCBE. RPL17A and ACTB genes were used as housekeeping genes. Data are shown as the mean ± S.E.M. of fold change (2^−(ΔΔCt)^) compared to the control group without treatment (*n* = 3, * *p* < 0.05, ** *p* < 0.01, *** *p* < 0.001, **** *p* < 0.0001, Student *t*-test.).

LOCBE at 5 µg/mL increased the expression of transcriptional factors and cytokine genes in macrophages after 6 h of treatment. As shown in Figure 3, LOCBE treatment significantly upregulates *STAT1*, *STAT3* and *NF-κB* expression. While *STAT1* was expressed at a 4.8 ± 1.19-fold higher rate when compared to the control, *STAT3* and *NF-κB* were expressed at a 2.55 ± 0.23 and 3.35 ± 0.64-fold higher rate, respectively. Interferon-regulatory factors, IRF4 and IRF5, were not affected (Figure 3A). LOCBE also induced an increase in proinflammatory cytokines gene expression, such as *IL-1β* (4.56 ± 0.36-fold higher), *IL-6* (289.9 ± 48.62-fold higher), and *IL-8* (4.8 ± 0.98-fold higher) (Figure 3B). Although the increase in *TNF-α* expression was only 1.55 ± 0.05-fold higher, this was statistically significant.

### 2.4. LOCBE Induces Inflammatory Cytokine and Chemokine Release

**Figure 4:**
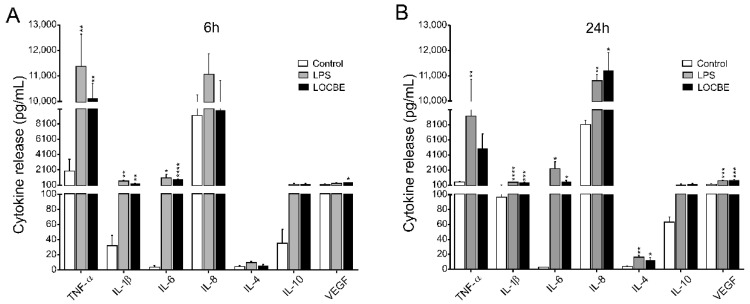
Cytokine secretion profile in LOCBE-treated macrophage. Cells were treated with 5 μg/mL LOCBE and cytokine release was evaluated after 6 h (**A**) and 24 h (**B**) by Millipore Multiplex assay. Data are shown as mean ± S.E.M. in pg/mL. Significance of the differences were evaluated with t Student test (* *p* < 0.05, ** *p* < 0.01, *** *p* < 0.001, **** *p* < 0.0001).

Macrophage cytokine and chemokine release profile was evaluated after 6 and 24 h of LOCBE treatment. As indicated in Figure 4 5 μg/mL LOCBE was able to directly induce the expression of proinflammatory cytokine in both treatment times. After 6 h of treatment, macrophages enhance the release of the cytokines TNF-α (1959 ± 512 vs. 9820 ± 623 pg/mL for control and LOCBE, respectively, *n* = 3, ** *p* < 0.01), IL-1β (32.1 ± 13.6 vs. 272.3 ± 44.3 pg/mL for control and LOCBE, respectively, *n* = 3, ** *p* < 0.01), IL-6 (3.9 ± 1.9 vs. 817.5 ± 86.5 pg/mL for control and LOCBE, respectively, *n* = 3, **** *p* < 0.0001), and VEGF (183 ± 54.9 vs. 498 ± 61.0 pg/mL for control and LOCBE, respectively, *n* = 3, * *p* < 0.05) (Figure 4A).

Moreover, after 24 h of LOCBE treatment, there was a significant liberation of all cytokines similar to 6 h treatment, such as IL-1β (96.4 ± 4.7 vs. 443.9 ± 41.23 pg/mL for control and LOCBE, respectively, *n* = 3, **** *p* < 0.0001), IL-6 (2.7 ± 0.4 vs. 538.7 ± 196.2 pg/mL for control and LOCBE, respectively, *n* = 3, * *p* < 0.05) and VEGF (217.4 ± 23.0 vs. 761.4 ± 74.34 pg/mL for control and LOCBE, respectively, *n* = 3, *** *p* < 0.001. In addition to these cytokines, after 24 h of LOCBE treatment, it was observed that there was an increase in the release of IL-8 (8132 ± 552.0 vs. 11,205 ± 718.7 pg/mL for control and LOCBE, respectively, *n* = 3, * *p* < 0.05), and IL-4 (3.9 ± 0.5 vs. 12.7 ± 3.2 pg/mL for control and LOCBE, respectively, *n* = 3, * *p* < 0.05) (Figure 4B). TNF-α and IL-10 release was not affected by the 24 h treatment of macrophages with LOCBE.

The chemokine profile (CXCL10, CCL22, CCL2, CCL3 and CCL4) in LOCBE-treated macrophages was also analyzed. Values and S.E.M for chemokine release are presented in Table 1. LOCBE was able to induce significant CXCL10 release after 6 h of treatment (11,888 pg/mL) in comparison with non-treated cells (219.8 pg/mL). On the other hand, the other chemokines showed a tendency to increase their expression after 6 h of LOCBE treatment, however not in a significant way. In contrast, after 24 h of treatment, an increased release of all chemokines analyzed were observed when compared to control group, CXCL10 (2670 pg/mL), CCL22 (114.1 pg/mL), CCL2 (455.6 pg/mL), CCL3 (450.9 pg/mL) and CCL4 (5161 pg/mL). Macrophages after 24 h of treatment with LOCBE showed an increase in CXCL10 (17,275 pg/mL), CCL22 (2283 pg/mL), CCL2 (6015 pg/mL). Regarding CCL3 and CCL4 release, the values detected after LOCBE treatment exceeded the maximum limit of the detection curve of the method used, which were 8200 and 8643 pg/mL, respectively.

### 2.5. LOCBE Increases Expression of Membrane Markers

**Figure 5:**
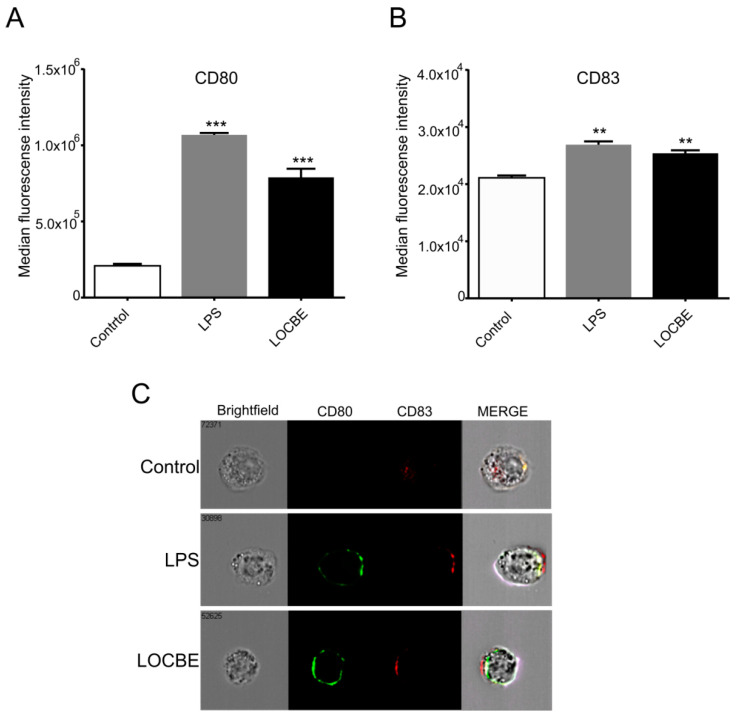
LOCBE increases the expression of macrophage activation markers. Macrophages were treated 24 h with LOCBE at 5 μg/mL, then stained with specific antibodies for CD80-BB515 (**A**) and CD83-APC (**B**) followed by Imaging flow Cytometry analysis. Data are shown as the mean of Median of Fluorescence Intensity (MFI) ± S.E.M. (**C**) Representative images of CD80 (green) and CD83 (red) staining. (** *p* < 0.01, *** *p* < 0.001).

Macrophages were treated with LOCBE at 5 μg/mL for 24 h to evaluate its effect on the polarization surface markers expression by flow cytometry. We observed that LOCBE upregulated the expression of costimulatory molecules, such as CD80 (Figure 5A) and CD83 (Figure 5B), in macrophages. Thus, LOCBE-treated macrophages presented values of MFI for CD80 of 7.9 × 10^5^ ± 5.7 × 10^4^ vs. 2.1 × 10^5^ ± 1.3 × 10^4^ for control, and values of MFI for CD83 of 2.5 × 10^4^ ± 5.2 × 10^3^ vs. 2.1 × 10^4^ ± 4.04 × 10^3^ for control. LPS at 1 μg/mL was used as the positive control.

## 3. Discussion

Envenomation by contact with *L. obliqua* caterpillars is characterized by an intense local inflammatory reaction initially manifested by pain and edema formation, which can later develop into a hemorrhagic disturbance and persistent systemic inflammatory reaction. In recent years, studies have tried to elucidate the molecular mechanism of those effects and shown the role of the inflammatory response in serious envenomation clinical complications, such as vascular disorders and renal dysfunction [22].

In this work, we investigated the proinflammatory effect on human THP-1-derived macrophages induced directly by LOCBE in vitro. Our data showed that the venom was able to enhance the secretion of proinflammatory cytokines and chemokines from macrophages and also increases macrophage activation surface marker expression. Furthermore, LOCBE induces NF-κB pathway activation and upregulates transcriptional factor genes, such as STAT1 and STAT3, which are described as important pathways to lead this cell to a proinflammatory phenotype.

In general, crude animal venoms have high cytotoxicity and capacity to induce cell death [35]. Our results show that LOCBE, at the concentrations assayed, has no cytotoxic effect or ability to decrease cellular viability in macrophages. In fact, studies have demonstrated that *L. obliqua* venom possesses proliferative effects and it is able to increase the viability of several cell lines, such as human glioblastoma and colon adenocarcinoma [36]. Several studies with isolated proteins from LOCBE, known as LOPAP, a prothrombin activator protease, and LOSAC, a Factor X activator, have shown them to promote cytoprotection in fibroblast, endothelial, neutrophil and neuron cells under nutrient deprivation through an antiapoptotic mechanism. Besides cytoprotection, LOSAC was also able to induce proliferative effects on vascular endothelium [16,26,37,38,39].

Macrophages are the major effector cells mediating the inflammatory response in damaged tissues. Depending on the external stimulus, it can polarize to a proinflammatory or anti-inflammatory phenotype expressing specific mediators and membrane markers. A common signaling event in macrophage polarization to a proinflammatory profile is the activation of the canonical NF-κB pathway. Once activated, p65 from NF-κB complex induces the transcription of IL-1β, TNF-α and IL-6 genes that are responsible for initiating the inflammatory response [40]. Our results show that a low dose of LOCBE is able to activate the NF-κB pathway in macrophages derived from THP-1 monocytes in a short period of time. Corroborating our findings, Nascimento-Silva and collaborators [20] showed that low non-hemorrhagic doses of *L. obliqua* venom are able to induce NF-κB activation in endothelial cells.

Given our observation that LOCBE activates the NF-κB pathway, we examined the mRNA levels of other transcription factors associated with inflammation and macrophage activation. mRNA levels of *STAT1*, *STAT3* and the p65 protein gene of the NF-κB complex were all upregulated. STAT1 is known to induce the expression of genes related to NO production, such as NOS2 and MHC II leading macrophages to M1 profile [41,42]. Meanwhile, STAT3 can be activated by IL-6 and also IL-10, and this leads to the upregulation of these two antagonist cytokines genes, serving as a regulator of an exacerbated proinflammtory response [43]. Simultaneously, we also showed that after 6 h of LOCBE treatment, the levels of *TNF-α*, *IL-1β*, *IL-6* and *IL-8* mRNA were also upregulated. Thus, our results suggest that NF-κB, and also STAT1 and STAT3, could lead these cells to a transcriptional cascade inducing the proinflammatory cytokine release.

Therefore, we investigated the levels of proinflammatory and anti-inflammatory cytokines and chemokines secreted by macrophages after 6 and 24 h of LOCBE treatment. The release of high concentrations of TNF-α, IL-1β, IL-6 was observed in the supernatants after 6 h of LOCBE treatment. Moreover, the amount of pro-inflammatory cytokines IL-1β, IL-6 and IL-8 released after 24 h was higher than after 6 h of treatment, suggesting a persistence of the inflammatory response. LOCBE also showed to induce an increase in the release of a chemokine panel in THP-1-derived macrophages. There was an initial effect on CXCL10 release after 6 h of treatment, and then, after 24 h, an increase in the release of CCL22, CCL2, CCL3 and CCL4 was observed. These chemokines play an important role in recruiting effector blood cells to the site of inflammation, where each type induces the recruitment of well-defined leukocyte subtypes. Interestingly, CXCL10 appears to play an important role in the first hours of treatment with LOCBE. This chemokine is related to the recruitment of Th cells and natural killer cells to the inflammation site [44].

On the other hand, we also observed an increase in anti-inflammatory mediators after 24 h of LOCBE treatment. IL-4 e VEGF, two anti-inflammatory cytokines and the chemokine CCL22. The production of anti-inflammatory cytokines can be activated by an exacerbated proinflammatory stimulus, as mechanism of these cells to induce M2 activation and suppress the proinflammatory response [32,43]. IL-4, for example, inhibits the macrophage production of proinflammatory cytokines, including TNF-α, IL-1, and IL-6 [45,46], while VEGF and CCL22 are described to be secreted by anti-inflammatory macrophages [47,48]. Additionally, our results show that IRF4 and IRF5 were not modulated by LOCBE-treatment. IRF5 regulates the M1 macrophage phenotype, whereas IRF4 regulates M2 polarization [49]. Thus, both transcription factors have non-redundant roles in LOCBE-induced effects in macrophages.

Aiming to characterize the macrophage activation, we observed an increase in the expression of the costimulatory molecules CD80 and CD83. Both proteins are responsible for assisting the activation of naive T lymphocytes, making these cells effectors of the immune response, since the cytokines produced by T lymphocytes are powerful inducers and regulators of inflammatory response [50]. All those results together indicate that LOCBE can directly activate macrophages and lead these cells to a proinflammatory phenotype.

Macrophages are important cells in the cross-talk between inflammation and homeostasis. The release of excessive proinflammatory cytokines by macrophages at the inflammation site leads to the disturbance of vascular tissue with an increase in NO production, which contributes to the increase in ROS and the modulation of vascular tone [51]. In endothelial cells specifically, these proinflammatory mediators cause an increase in the expression of COX-2 and the production of prostaglandins and NADPH-oxidase, causing increased permeability of the vessels, facilitating the infiltration of fluids in the tissue and the formation of edema [52,53].

In the past few years, many studies have shown that LOCBE and its toxins cause proinflammatory effects in vivo and in vitro. Studies showed that LOCBE direct induces a proinflammatory phenotype in endothelial cells where LOCBE increases adhesion molecules, metalloproteinases (MMPs), COX-2, IL-6, and IL-8 expression in these cells [14,20]. Moreover, the LOPAP protein has been shown to increase the release of IL-8 in fibroblasts [25]. Furthermore, an increase in ROS dependent on NADPH expression was also seen in vascular smooth muscle cells treated with *L. obliqua* venom [24]. Moreover, studies in vivo using a model of LOCBE-induced acute renal failure pointed out that kidneys of envenomed rats presented increased levels of superoxide, NO, MMPs and an excessive amount of proinflammatory cytokines such as TNF-α and IL-1β [22]. In parallel, Barrios and colleagues [54] demonstrated an increase in the release of TNF-α and NO after 1 h of administration of *Lonomia Achelous* venom in the blood of animal models. This other species of caterpillar of the genus Lonomia triggers hemorrhagic events physiologically similar to *L. obliqua* poisoning.

There are reports in the literature demonstrating that whole venoms and toxins isolated from snakes and scorpions, for example, are able to activate macrophage functions, such as phagocytosis and the production of reactive oxygen species, cytokines and eicosanoids [55,56,57,58]. *Bothrops alternatus* snake venom is able to induce a proinflammatory reaction in vivo and increase IL-1, IL-12, TNF-α and COX2 in murine macrophages in vitro [58]. Scorpion venoms such as *Tityus serrulatus* and *Androctonus australis* Hector are also efficient inducers of macrophage TNF-α secretion [59,60]. Those results are related to the formation of prominent local edema, pain, and extensive swelling in snake-envenomed victims and play an important role in the genesis of organ failure during severe scorpion envenomation.

In the case of lonomism, once the venom is injected in the victim, the first clinic manifestation is a proinflammatory reaction, and the results shown here suggest that macrophages can be participating as an effector of those events. Since LOCBE can directly induce these cells to release a considerable amount of proinflammatory mediators, macrophages might contribute to endothelium activation and the maintenance of the systemic inflammatory reaction observed in the victim together with the hemorrhagic events. An excess of proinflammatory mediators increases endothelium permeability and it can contribute to hemorrhagic events mainly in the microvascular vases in the brain that are the most cases of fatal outcomes after the envenomation. Furthermore, an exacerbated proinflammatory mediator release was suggested to be related to the renal failures in animal models.

In summary, the data demonstrate that *L. obliqua* venom has a direct proinflammatory effect on macrophages derived from the THP-1 lineage, promoting a high release of cytokines and chemokines and a change in intracellular signaling cascades that result in the activation of these cells to a pro-inflammatory profile. Here, we also showed that the NF-κB pathway plays an important role in inducing this phenotype. Toxins, such as LOPAP, isolated from the LOCBE have already been characterized with regard to some of their effects on the modulation of molecules, with an important role in the inflammatory process. These and other venom components may be responsible for the effect mentioned.

## 4. Conclusions

*Lonomia obliqua* venom directly induces macrophage to take on a proinflammatory activation phenotype, increasing cytokine release and upregulating costimulatory cell-surface markers, possible with the involvement of NF-κB pathway that is responsible for inducing the expression of many inflammatory cytokine and chemokine genes.

## 5. Materials and Methods

### 5.1. Lonomia obliqua Crude Bristle Extract Obtention

*Lonomia obliqua* crude bristle extract (LOCBE) was obtained from caterpillars collected in the south of Brazil (states of Santa Catarina, Rio Grande do Sul and Paraná) and provided by the Butantan Institute. Briefly, to obtain the extract, caterpillar’s bristles were harvested by cutting them at their base, ground in a mortar and homogenized in PBS (pH 7.4). The solution was sterilized by filtration using Millex filters (Millipore, Darmstadt, Germany, #SLGV013SL). Protein content was evaluated using the Pierce BCA Protein Assay Kit (Thermo Scientific, Rockford, IL, USA), according to the manufacturer’s protocol, and stored at −80 °C until use.

The endotoxin levels present in LOCBE samples were assessed following Good Manufacturing Practice (GMP) using the Gel Clot—limit test assay. Endotoxin was detected at an acceptable level between 0.1 and 1.2 EU/mL at 5 μg/mL LOCBE. LPS 1 μg/mL (99% purity) was employed as a positive control.

### 5.2. Cell Culture and Differentiation

THP-1 cell line was obtained from the American Type Culture Collection—ATCC (Manassas, VA, USA). The THP-1 monocytes were cultured in complete culture medium composed by RPMI 1640 medium (Sigma-Aldrich, St. Louis, MO, USA, #R6504) supplemented with 10% fetal bovine serum (Gibco, Grand Island, NY, USA, #26140079), 100 U penicillin/streptomycin (Gibco, Grand Island, NY, USA, #15140122), 2 mM L-glutamine (Sigma-Aldrich St. Louis, MO, USA, #A2916801), 1 mM Sodium Pyruvate (Sigma-Aldrich, Sigma-Aldrich, St. Louis, MO, USA, #P5280) and incubated at 37 °C in 5% CO_2_. THP-1 monocytes cells were differentiated to macrophages using phorbol 12-myristate 13-acetate (PMA) (Sigma-Aldrich, St. Louis, MO, USA, #P8139). Briefly, 25 nM PMA was added to a medium culture containing 2 × 106 cells in a T25 cell culture flask for 48 h, followed by 24 h of rest in a PMA free medium, as previously suggested to inflammatory studies [61]. Monocyte differentiation into macrophages was verified by both total cell adhesion to the plate and morphology and membrane marker expression, CD14 and CD11b (Supplementary Figure S1).

### 5.3. Cellular Viability and Cytotoxicity

THP-1 macrophage viability was evaluated by the MTT colorimetric assay [62]. After 48 h with PMA, macrophages were detached using 1 mL of TripleExpress (Gibco, Grand Island, NY, USA, #12604021) for 5 min, then cells were seeded in a 96-well plate at 2 × 104 cells/well. After 24 h resting in PMA-free medium, cells were incubated with LOCBE at 5, 25, and 50 µg/mL, 1 µg/mL LPS (Sigma-Aldrich, St. Louis, MO, USA, #L4516) or 1 mM H_2_O_2_ (Sigma-Aldrich, St. Louis, MO, USA, #323381) for 24 h. The cells were incubated with 100 µL of MTT (Sigma-Aldrich, #M2003) solution (0.5 mg/mL in complete culture medium) at 37 °C for 3 h. MTT formazan crystals were solubilized in 100 µL of DMSO (Sigma-Aldrich, St. Louis, MO, USA, #D8418) during 5 min. Absorbance was read at 540 nm using a spectrophotometer Spectra Max 190 (Molecular Devices, San José, CA, USA). The percentage of LOCBE-induced cell death was determined from the experimental group absorbance divided by untreated controls absorbance × 100%.

Lactate dehydrogenase (LDH) release was quantified in 50 µL supernatants using the Cytotox 96 NonRadioactive Cytotoxicity assay (Promega, Madison, WI, USA, #G1780) following the manufacturer’s instructions. Values were expressed as the percentage of maximum LDH release (set at 100%) obtained through total lysis of cultured cells.

### 5.4. NF-κB Pathway Activation

High-content screening (HCS) was used to detect data on multiple parameters in single cells as well as in populations of cells [63], aiming to measure the *L. obliqua* venom capacity to induce NF-κB pathway activation by measuring the protein translocation from the cytoplasm to the nuclei. After differentiation, macrophages were seeded in 96-well microplates for fluorescence detection (Greiner, Kremsmünster, Austria) at 2 × 10^4^ cells/well and incubated for 24 h with PMA-free medium. Macrophages were treated with LOCBE at 5, 25 and 50 μg/mL for 30 min and with 1 μg/mL LPS as a positive control. Afterwards, cells were fixed with 100 µL PHEM buffer (2 nM HEPES, 10 mM EGTA, 2 mM MgCl_2_, 60 mM PIPES at pH 6.9) containing 4% paraformaldehyde (PFA) (Sigma-Aldrich, St. Louis, MO, USA, #P6148) for 1 h, followed by permeabilization with 0.5% Triton-100 (Sigma-Aldrich, St. Louis, MO, USA, #T8787) in PHEM buffer for 5 min. Macrophages were washed 3× with Glycine 0.1 M in PHEM buffer before blocking with 1% BSA (Sigma-Aldrich, St. Louis, MO, USA, #A7606) in PHEM buffer for 30 min. The cells were stained overnight with primary anti-NF-kB (p65) antibody (Cell Signaling, Danvers, MA, USA, #3033S) (1:500) at 4 °C. Plate was washed with PHEM buffer and then stained for 1h at room temperature with goat anti-rabbit AlexaFluor 647 antibody (ThermoFisher Scientific, Bengaluru, India, #A27040) (1:1000) and Hoechst 33342 (Thermo Fisher Scientific, Eugene, OR, USA, #62249) at 5 μM for labeling DNA.

Images were obtained using ImageXpress Micro Confocal High-Content Imaging System (Molecular Devices, San Jose, CA, USA). To determine NF-κB translocation from the cytoplasm to nucleus, we analyzed fluorescence intensity in the nucleus area and intensity in the cytoplasm area (Inner/Outer Intensity Ratio). The translocation value (%) was calculated by the ratio of nuclear and cytoplasmic region fluorescence intensity (Nuc/Cyt Ratio) (Correlation coefficient ≥0.6). In this case, the module “Translocation Enhanced” was used. Three independent experiments were performed, and the percentage of positive cells was calculated using the average of 16 sites/well.

### 5.5. Gene Expression

For gene expression analysis, THP-1 derived macrophages were treated with LOCBE at 5 µg/mL for 6 h. After treatment, supernatants were collected and stored at −80 °C for cytokine analyses, and cells were washed three times with PBS and the pellet was stored at −80 °C. Total RNA was isolated and purified from differentiated cells using the RNAspin Mini kit (GE Healthcare, Chicago, IL, USA) according to the manufacturer’s instructions applying an additional treatment with DNase I for 1 h. Total RNA was quantified using the ND-1000 NanoDrop (Thermo Fisher, Wilmington, DE, USA). Real-time qPCR was performed to analyze transcriptional factors and their responsive genes expression. First, reverse transcription was performed to obtain cDNA followed by qPCR. For all genes, oligo-dT and random primed reverse transcription was performed using 500 ng of total RNA in 20 µL of RT reaction with SuperScript III (Invitrogen, Carlsbad, CA, USA), followed by qPCR using 2 µL of the 10-fold diluted RT reaction in 8 µL of qPCR (QuantStudio 3 Real-Time PCR System, ThermoFisher Scientific). Transcript levels were normalized with Ct average from RPL17A and ACTB genes, and represented as relative abundance using the delta Ct method [64]. Conditions for PCR reactions were: 40 cycles of 95 °C/15 s, 60 °C/1 min, using specific primers to measure the expression of a panel of genes including *NF-κB*, *STAT1*, *STAT3*, *IRF4* and *IRF5* transcriptional factors and *TNF-α*, *IL-1β*, *IL-6* and *IL-8* cytokines.

### 5.6. Cytokine and Chemokine Profile Liberation

Cell-free supernatants were used to determine cytokine and chemokine secretion after 6 and 24 h after LOCBE treatment. The quantification was performed by multiplex analysis using the Milliplex MAP Human Cytokine/Chemokine Magnetic Bead Panel (Millipore, #HCYTOMAG-60K-13) with detection and analysis using the Luminex-200 system (Millipore). The software for acquisition was Luminex xPONENT 4.3 and for results analysis we used the MILLIPLEX Analyst 5.1. The assay kit was performed according to the manufacturer’s specification. The range of detection for each molecule (TNF-α, IL-1β, IL-6, IL-8, IL-4, IL-10, VEGF, CXCL10, CCL22, CCL2, CCL3 and CCL4) was in pg/mL.

### 5.7. Cell Surface Proteins Expression

After differentiation protocol, 2 × 106 macrophages were treated with LOCBE at 5 μg/mL for 24 h. Then, supernatants were collected and stored at –80 °C for cytokine analyses, and cells were detached, washed with PBS, and fixed with 0.25% PFA in PBS for 1 h at room temperature. Macrophages were washed three times with PBS and stained with anti-CD80 conjugated with BB515 and anti-CD83 conjugated with APC (BD Biosciences, Franklin Lakes, NJ, USA, #565058 and #551073, respectively) antibodies (1:50) for 2 h at room temperature. Samples were analysed on Amnis ImageStreamX MkII flow cytometer using ISX software (Luminex Corporation, Austin, TX, USA) equipped with 4 lasers (405, 488, 642 and 785 nm (SSC). Experiments were carried out using 488 and 642 lasers set to maximum power, and all data were acquired with 40× magnification.

The BB515 signal was collected in channel 2 (480–560 nm filter) and APC signals in channel 11 (660–745 nm filter). Channels 1 (430–480 nm filter) and 9 (570–595 nm filter) were used as brightfield channels and channel 6 (745–800 nm filter) for SSC detection. At least 10,000 events were acquired. All data were analyzed using IDEAS^®^ Software.

### 5.8. Statistical Analysis

All experiments were carried out at least in at least three independent biological experiments and data were recorded as mean ± standard error of the mean. Comparison between groups was achieved using non-parametric Student’s *t*-tests using Prism 6.01 software (GraphPad Software). Significance was set at *p* < 0.05.

## Figures and Tables

**Table 1:** Macrophage upon treatment with LOCBE chemokine release profile.

Analyte (pg/mL)	Control		LPS		LOCBE	
	6 h	24 h	6 h	24 h	6 h	24 h
**CXCL10**	219.8 ± 101	2670 ± 200.3	13,831 ± 2379 **	16,729 ± 503.3 ****	11,888 ± 1362 **	17,275 ± 107.6 ****
**CCL22**	17.53 ± 1.2	114.1 ± 23.1	69.20 ± 15.7 *	2405 ± 612.6 *	36.23 ± 12.5	2283 ± 263.5 ***
**CCL2**	5810 ± 1826	455.6 ± 51.9	8678 ± 341.5	8448 ± 217.3 ****	7399 ± 979.9	6015 ± 1471 *
**CCL3**	5587 ± 1329	450.9 ± 110	7887 ± 312.8	8108 ± 92.5 ****	7294 ± 732	8200↑
**CCL4**	5802 ± 1462	5161 ± 784.3	8643↑	8643↑	8090 ± 553.5	8643↑

Chemokine secretion profile in *Lonomia obliqua* bristle extract-treated macrophage. Cells were treated with 5 μg/mL of LOCBE for 6 and 24 h and evaluated for the release of CXCL10, CCL22, CCL2, CCL3 and CCL4 by Multiplex assay. Data are represented in pg/mL with mean ± SEM. Values above the detection limit of the assay are indicated by a vertical arrow (↑). Significant differences in relation to untreated control were evaluated using Student’s *t* test (* *p* < 0.05, ** *p* < 0.01, *** *p* < 0.001, **** *p* < 0.0001).

## Data Availability

The data presented in this study are available on request from the corresponding author.

## Supplementary Materials

The following are available online at https://www.mdpi.com/article/10.3390/toxins13070462/s1, Figure S1: THP-1 monocyte differentiation in macrophages. The differentiation protocol wasevaluated by image flow cytometry with anti-CD14-APC and anti-CD11b-PECy7 antibodies, and cellular morphology was assessed by confocal microscopy.
