# Supplementary material for: Lonomia obliqua Venom Induces NF-κB Activation and a Pro-Inflammatory Profile in THP-1-Derived Macrophage
Source: Toxins (Basel). 2021 Jun 30;13(7):462. doi: 10.3390/toxins13070462 (PMC8309978; doi:10.3390/toxins13070462)
Supplement: Supplementary file 1 [file toxins-13-00462-s001.zip › toxins-1255794-supplementary corrected.pdf]

Supplementary Materials: *Lonomia obliqua* Venom Induces NF-κB Activation and a Pro-Inflammatory Profile in THP-1-Derived Macrophage

Douglas Oliveira, Jean Gabriel de Souza, Miryam Paola Alvarez-Flores, Priscila Cunegundes, Carlos DeOcesano-Pereira, Aline Maia Lobba, Renata N. Gomes and Ana Marisa Chudzinski-Tavassi

THP-1 monocyte cells differentiated in macrophages

After the differentiation protocol, macrophage obtention was confirmed by CD14 and CD11b expression modulation and cellular morphology. Figure S1 A and B show a significant increase in both CD14 and CD11b expression in macrophages accessed by imaging flow cytometry. CD14 presented a mean of 1.2 × 104 in monocytes and 3.3 × 104 in macrophages, while CD11b was 3.4 × 104 in monocytes and 9.8 × 104 in after differentiation. Data are shown as the median of fluorescence intensity ($p < 0.05$). We also evaluate macrophage adherence and morphology. Macrophages were differentiated in microscope cover glass, and then these cells and monocytes were fixed and stained with phaloidin-AF488 (1:1000) and Hoechst (5 µM). Image capture was performed using a Zeiss LSM 510 confocal microscope and processed by AxioVision 4.7.2 software. Cells after differentiation presented total adherence and an increase in size and cytoplasmic volume when compared to THP-1 monocytes.

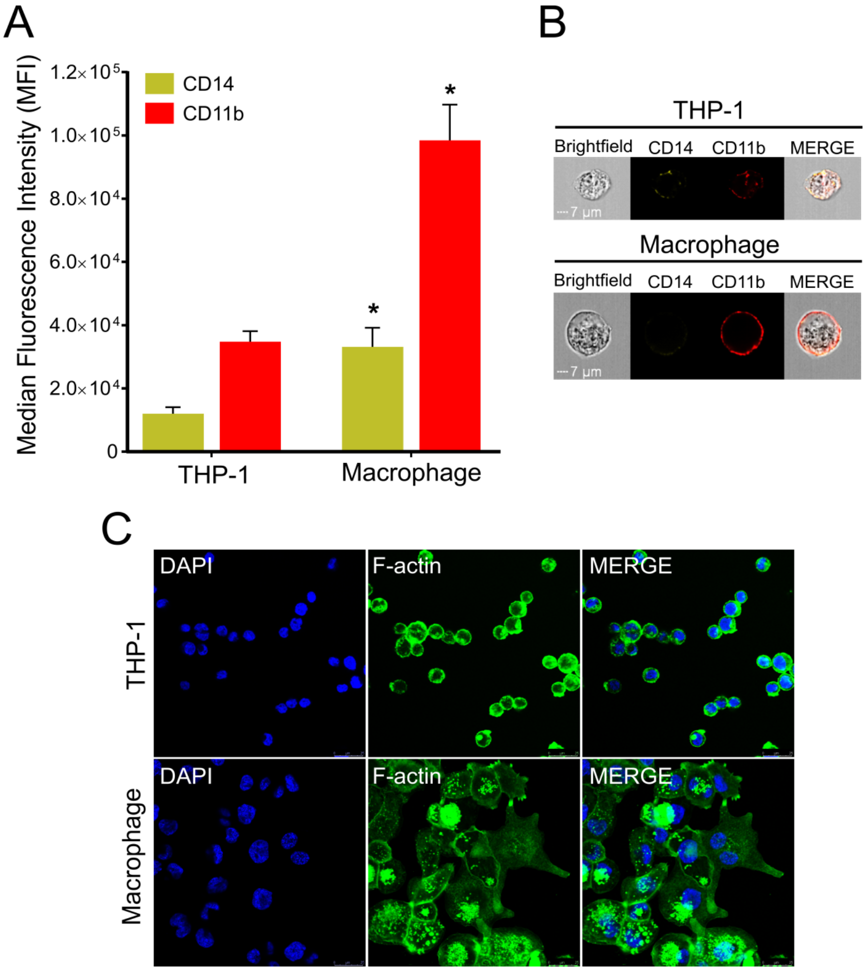

 https://doi.org/10.3390/toxins13070462

Figure S1. THP-1 monocyte differentiation in macrophages. The differentiation protocol was evaluated by image flow cytometry with anti-CD14-APC and anti-CD11b-PECy7 antibodies, and cellular morphology was assessed by confocal microscopy. (**A**) Upregulation of membrane markers after PMA treatment measured by fluorescence intensity (* $p < 0.05$). (**B**) Representative cytometry images of CD14 (red) and CD11b (yellow) staining. (**C**) Monocytes' and macrophages' morphological differences with phalloidin (green) and nuclei (blue).
